# Ventilator-associated pneumonia and ICU mortality in severe ARDS patients ventilated according to a lung-protective strategy

**DOI:** 10.1186/cc11312

**Published:** 2012-04-18

**Authors:** Jean-Marie Forel, François Voillet, Daniel Pulina, Arnaud Gacouin, Gilles Perrin, Karine Barrau, Samir Jaber, Jean-Michel Arnal, Mohamed Fathallah, Pascal Auquier, Antoine Roch, Elie Azoulay, Laurent Papazian

**Affiliations:** 1 Service de Réanimation des Détresses Respiratoires et Infections Sévères, Assistance Publique Hôpitaux de Marseille, URMITE CNRS-UMR 6236, Aix-Marseille Univ, Marseille, 13015, France; 2 Service de Réanimation Médicale, Hôpital Pontchaillou, Rennes, 35033, France; 3 Service de Réanimation des Urgences, Assistance Publique Hôpitaux de Marseille, Marseille, 13005, France; 4 Laboratoire de Santé Publique, Faculté de Médecine, Marseille, Aix-Marseille Univ, Marseille, 13005, France; 5 Service de Réanimation Chirurgicale, Hôpital Saint Eloi, Montpellier, 34295, France; 6 Service de Réanimation polyvalente, Hôpital Font-Pré, Toulon, 83100, France; 7 Centre d'Investigations Cliniques, Assistance Publique Hôpitaux de Marseille - INSERM 9502, Marseille, 13385, France; 8 Service de Réanimation Médicale, Hôpital Saint-Louis, Paris, 75010, France

## Abstract

**Introduction:**

Ventilator-associated pneumonia (VAP) may contribute to the mortality associated with acute respiratory distress syndrome (ARDS). We aimed to determine the incidence, outcome, and risk factors of bacterial VAP complicating severe ARDS in patients ventilated by using a strictly standardized lung-protective strategy.

**Methods:**

This prospective epidemiologic study was done in all the 339 patients with severe ARDS included in a multicenter randomized, placebo-controlled double-blind trial of cisatracurium besylate in severe ARDS patients. Patients with suspected VAP underwent bronchoalveolar lavage to confirm the diagnosis.

**Results:**

Ninety-eight (28.9%) patients had at least one episode of microbiologically documented bacterial VAP, including 41 (41.8%) who died in the ICU, compared with 74 (30.7%) of the 241 patients without VAP (*P *= 0.05). After adjustment, age and severity at baseline, but not VAP, were associated with ICU death. Cisatracurium besylate therapy within 2 days of ARDS onset decreased the risk of ICU death. Factors independently associated with an increased risk to develop a VAP were male sex and worse admission Glasgow Coma Scale score. Tracheostomy, enteral nutrition, and the use of a subglottic secretion-drainage device were protective.

**Conclusions:**

In patients with severe ARDS receiving lung-protective ventilation, VAP was associated with an increased crude ICU mortality which did not remain significant after adjustment.

## Introduction

Acute respiratory distress syndrome (ARDS) still carries a high mortality rate. Ventilator-associated pneumonia (VAP) is a complication of ARDS that may increase the risk of multiple organ failure and death. However, the impact of nosocomial pneumonia on mortality in mechanically ventilated patients remains controversial [1-4], and few data are available on mortality in ARDS patients who experience VAP [5-10]. Moreover, many of the studies related to VAP in ARDS patients were performed before the use of lung-protective mechanical-ventilation strategies. Modifications in mechanical-ventilation settings may have modified the epidemiology and outcomes of VAP in patients with severe ARDS [11].

Here, we report the results of an epidemiologic study performed in all patients included in a multicenter randomized, placebo-controlled double-blind trial conducted to determine whether briefly administering the neuromuscular blocking agent (NMBA) cisatracurium besylate early in the course of severe ARDS improved the clinical outcomes. The results of this therapeutic intervention have been reported elsewhere [12]. The main objective of this prospective study was to determine whether bacterial VAP complicating severe ARDS was associated with ICU death in patients receiving lung-protective mechanical ventilation. We also determined the incidence and risk factors of VAP.

## Materials and methods

Patients were enrolled from March 2006 through March 2008 at 20 French intensive care units [12]. The study protocol was approved for all centers by the ethics committee of the Marseille University Hospital (Comité Consultatif de Protection des Personnes dans la Recherche Biomédicale Marseille 1, no 04/37). According to French law, written informed consent was obtained from the patients or their surrogates.

### Patients

Patients receiving endotracheal mechanical ventilation for severe ARDS were eligible if the following criteria were met for no more than 48 hours before enrollment: ratio of partial pressure of arterial oxygen to fraction of inspired oxygen (PaO_2_:FIO_2_) no greater than 150 mm Hg at the time of enrollment, with a PEEP of at least 5 cm H_2_O and a tidal volume of 6 to 8 ml/kg, recent appearance of bilateral pulmonary infiltrates consistent with edema, and no evidence of left atrial hypertension.

Exclusion criteria were age younger than 18 years, lack of consent, continuous infusion of NBMA, known allergy to NMBA, known pregnancy, participation in another trial within 30 days before meeting the eligibility criteria, increased intracranial pressure, severe chronic respiratory disease requiring long-term oxygen therapy or home mechanical ventilation, actual body weight exceeding 1 kg/cm of height, severe burns, severe chronic liver disease (Child-Pugh class C), bone marrow transplantation or chemotherapy-induced neutropenia, pneumothorax, expected duration of mechanical ventilation shorter than 48 hours, and decision to withhold life-sustaining treatment.

### Design

Suspected pneumonia was defined as the presence of new or persistent radiographic features suggesting pneumonia without any other obvious cause and with two of the following: fever > 38°C, leukocytosis (> 11.0 10^9^/L) or leukopenia (< 3.5 10^9^/L), purulent endotracheal aspirate, recent isolation of pathogenic bacteria from the endotracheal aspirate [13], and increasing oxygen requirements. These criteria had to be present more than 2 days after ARDS onset. Bronchoalveolar lavage (BAL) was done in the affected region of the lung identified on a chest radiograph. When PaO_2_:FIO_2 _was lower than 80, protected minibronchoalveolar lavage with a protected telescopic catheter could be performed.

### Diagnosis of ventilator-associated pneumonia

In each ICU, an investigator made daily rounds to identify eligible patients and patients meeting criteria for suspected VAP. VAP was diagnosed when a quantitative culture of BAL fluid grew at least one bacterial organism in a concentration ≥ 10^4 ^colony-forming units (CFU)/ml or when mini-BAL fluid grew at least one bacterial organism in a concentration ≥ 10^3 ^CFU/ml [14].

### Definitions

Early-onset VAP complicating ARDS was defined as pneumonia diagnosed between the third and seventh days after ARDS onset. Late-onset VAP complicating ARDS was defined as pneumonia diagnosed more than 7 days after ARDS. Patients were monitored for VAP until their discharge from the ICU to a maximum of 90 days.

Empiric antibiotic therapy was started within a 24-hour period after microbiologic samples were obtained by BAL or protected telescoping catheter. Appropriate empiric antibiotic therapy was defined as *in vitro *susceptibility to at least one antibiotic of the organism(s) recovered from the BAL or the protected telescoping catheter samples. However, if *Pseudomonas *sp. was isolated, susceptibility to two drugs was required [15].

In patients with clinical and radiographic evidence of deterioration after an initial improvement after a first VAP, relapsing or recurrent infection was suspected if the organism found initially was identified, and superinfection, if a different organism was found.

### Data collection

Demographic data were collected. For the 24 hours before randomization, we collected the physiological variables, relevant therapeutic interventions, radiographic findings, co-morbidities, and medications. Ventilator settings, physiological variables, radiographic findings, and relevant therapeutic interventions were recorded just before starting the study drug and then 24, 48, 72, and 96 hours later, and daily thereafter between 6 a.m. and 10 a.m. until day 90 or hospital discharge while breathing without assistance. Patients were monitored daily for 28 days for signs of failure of nonpulmonary organs and systems [16]. We recorded the culture results, antibiotics used, duration of mechanical ventilation, ICU-stay length, and hospital-stay length. Throughout the ICU stay and until weaning off the ventilator for patients without VAP or the diagnosis of VAP for patients with VAP, we recorded daily the following factors potentially associated with VAP: neuromuscular blocking agent (NMBA) use during the first 48 hours, enteral nutrition, stress-ulcer prophylaxis, tracheostomy, transport out of the ICU (operating room, CT-scan), subglottic secretion drainage, selective digestive decontamination, emergency reintubation, prone positioning, and renal replacement therapy.

### Ventilation strategy

The volume-assist control mode was used, with a low tidal volume of 6 ml/kg of predicted body weight. The oxygenation goal was to maintain an arterial oxyhemoglobin saturation measured by pulse oximetry of 88% to 95% or a PaO_2 _of 55 to 80 mm Hg. This goal was achieved by adjusting FIO_2 _and PEEP, as done in the ARMA study [16]. The same ventilator-weaning protocol was used in both groups. Weaning was to be started on day 3 if the FIO_2 _was no greater than 0.6.

### Statistical analysis

We report means (± SD), relative risks with 95% confidence intervals (CIs), and medians with interquartile ranges (IQR) as appropriate. Differences between groups were assessed by using the Student *t *test, Wilcoxon test, χ^2 ^analysis, or Fisher Exact test. We used the Wilcoxon test to compare the numbers of ventilator-free days, ICU-free days, and organ-failure-free days, all of which had skewed distributions. All reported *P *values are two-sided.

Kaplan-Meier curves were constructed to assess the time from enrollment to death and to unassisted breathing within 90 days.

To analyze the effect of VAP on ICU mortality, we reported crude ICU mortality. We also performed a logistic regression (forced-entry model) to identify factors independently associated with ICU death. We planned to include all variables with *P *values < 0.20 by univariate analysis. Colinearity also was assessed. The occurrence of a VAP and the use of NMBA during the first 48 hours of ARDS were also entered in the model. In addition, we performed a multistate approach (clock-forward multistate model) to take into account both the time-dependence of the VAP and the presence of competing risks (for example, ICU death and discharge alive from the ICU) at each time point.

To identify risk factors for bacterial VAP, we also used the clock-forward multistate model. Of the 11 variables finally included in the model, seven had *P *values < 0.20 by univariate analysis (male sex, baseline Glasgow Coma Scale score, emergency reintubation, tracheostomy, transport out of the ICU, enteral nutrition, and subglottic secretion drainage) and four were predefined covariates (baseline SAPS II score, baseline PaO_2_:FIO_2_, baseline respiratory system compliance, and continuous NMBA use during the first 48 hours of ARDS).

All statistical analyses were performed by using PASW Statistics software version 17 and R development Core Team (2010). The multistage analysis was done by using mstate package 0.2.6 (see Additional file 1) [17,18].

## Results

### Study population

Table 1 reports the main characteristics of the 339 severe ARDS patients. ARDS was caused by a direct lung injury in 265 (78%) patients. The ICU mortality rate was 33.9% (115 of 339). We found no clinically significant differences between the groups with and without VAP regarding the main baseline characteristics, co-morbidities, or severity scores at ICU admission, except for a significantly higher proportion of male patients in the VAP group.

**Table 1:** Characteristics of the 339 patients with severe acute respiratory distress syndrome

	VAP *n *= 98	No VAP *n *= 241	*P* value
Age, mean ± SD	60 ± 14	57 ± 16	0.10
Males, *n *(%)	79 (81%)	159 (66%)	0.009
Tidal volume (ml/kg predicted body weight), mean ± SD^a^	6.34 ± 0.95	6.59 ± 1.1	0.04
Minute ventilation (liters/minute), mean ± SD^a^	10.4 ± 2.4	9.9 ± 2.3	0.06
PEEP applied (cm of water), mean ± SD^a^	9.2 ± 3.3	9.2 ± 3.4	0.97
Plateau pressure (cm of water), mean ± SD^a^	25.4 ± 4.8	24.4 ± 4.8	0.11
Respiratory system compliance (ml/cm of water), mean ± SD^a^	30.2 ± 9.7	32.0 ± 11.1	0.15
FiO_2_, mean ± SD^a^	0.77 ± 0.19	0.79 ± 0.19	0.43
pH, mean ± SD^a^	7.31 ± 0.10	7.32 ± 0.10	0.73
PaO_2_:FiO_2_, mean ± SD^a^	106 ± 33	111 ± 40	0.24
PaCO_2 _(mm Hg), mean ± SD^a^	47 ± 11	47 ± 11	0.66
SAPS II on admission, mean ± SD	50 ± 15	47 ± 15	0.19
SOFA on admission, mean ± SD	9.3 ± 3.8	9.3 ± 3.7	0.99
Glasgow Coma Scale score on admission, mean ± SD	10.9 ± 4.9	11.8 ± 4.2	0.08
Karnofsky score, mean ± SD	86 ± 14	88 ± 16	0.43
McCabe nonfatal, *n *(%)	74 (76%)	184 (76%)	0.99
Patients with immunodepression, *n *(%)			
No	85 (87%)	192 (80%)	0.13
Chemotherapy	2 (2%)	20 (8%)	
Long-term corticosteroids	4 (4%)	18 (7%)	
HIV	2 (4%)	0 (0)	
Other	3 (3%)	11 (5%)	
Main reason for ICU admission, *n *(%)			
Medical	68 (68%)	174 (72%)	0.60
Surgical emergency	18 (18%)	40 (17%)	0.75
Scheduled surgery	12 (12%)	27 (11%)	0.85
Corticosteroids for septic shock, *n *(%)^a^	40 (40%)	103 (43%)	0.81
Four radiologic quadrants involved, *n *(%)^a^	80 (82%)	196 (81%)	0.75
Direct lung injury, *n *(%)^a^	79 (81%)	186 (77%)	0.56
Primary cause of ARDS, *n *(%)^a^			NS
Community-acquired pneumonia	33 (34)	97 (40)	
Nosocomial pneumonia	21 (21)	36 (15)	
Ventilator-associated pneumonia	6 (6)	8 (3)	
Aspiration pneumonia	18 (18)	44 (18)	
Lung contusion	2 (2)	2 (1)	
Near-drowning	1 (1)	1 (0.5)	
Smoke inhalation	0 (0)	1 (0.5)	
Intraabdominal sepsis	7 (7)	23 (10)	
Sepsis other	3 (3)	10 (4)	
Acute pancreatitis	3 (3)	7 (3)	
Multiple transfusion	3 (3)	3 (1)	
Multiple trauma	2 (2)	2 (1)	
Shock	5 (5)	11 (5)	
Other	5 (5)	10 (4)	

^a^On inclusion. PEEP, positive end-expiratory pressure; SAPS II, Simplified Acute Physiology Score; SOFA, sequential organ-failure assessment; VAP, ventilator-associated pneumonia.

### Incidence of ventilator-associated pneumonia

During the study period, 120 suspected first episodes of bacterial VAP were evaluated by using BAL or mini-BAL. A bacterial VAP was diagnosed in 98 (28.9%) patients, with 26 cases of early-onset and 72 of late-onset first VAP episode. In 69 patients, a single VAP episode occurred. Twenty-two of the 98 patients had a second episode of VAP, whereas five had three episodes, and two had four episodes. A relapse or recurrent infection was diagnosed in six patients, and superinfections, in 32 patients.

**Figure 1:**
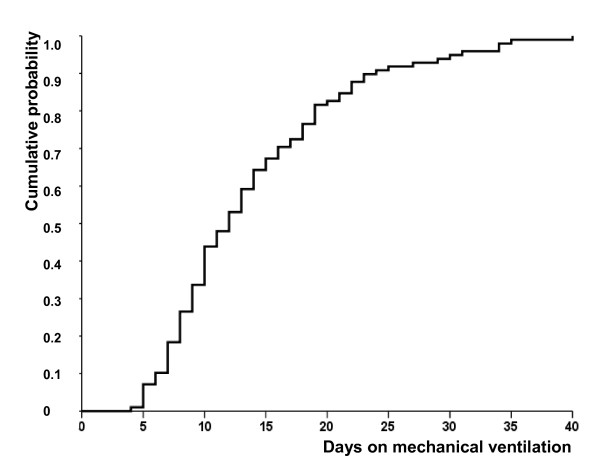
**Kaplan-Meier estimates of the cumulative probability of developing ventilator-associated pneumonia according to the number of days on mechanical ventilation in the group of 98 patients with severe acute respiratory distress syndrome who developed a ventilator-associated pneumonia**.

**Figure 2:**
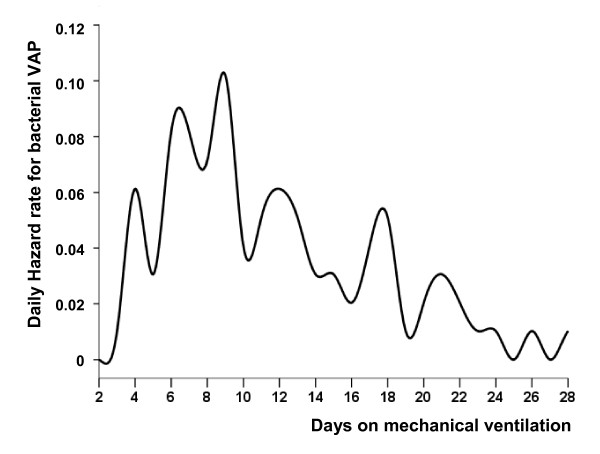
**Hazard rate for ventilator-associated pneumonia over time after the diagnosis of severe acute respiratory distress syndrome**. The hazard function evaluates the conditional probability of ventilator-associated pneumonia on the next day in an event-free patient. The hazard rate is the event rate per day over the duration of mechanical ventilation.

Median time on mechanical ventilation from ARDS onset to the first VAP episode was 10 days (IQR, 6 to 17 days). Median time on mechanical ventilation from endotracheal intubation to the first VAP episode was 11 days (IQR, 7 to 17 days). Most of the first VAP episodes occurred within 3 weeks after ARDS onset. Figure 1 shows the cumulative probability of developing VAP over time. The daily risk for developing bacterial VAP increased until day 9 and then decreased throughout the remainder of the ICU stay (Figure 2). The daily risk was 9% on day 7, 6% on day 12, and 3% on day 21.

In all, 112 bacterial strains grew in significant concentrations in BAL or protected mini-BAL specimens during the first VAP episode. As indicated in Table 2, the most common bacteria were nonfermenting, gram-negative bacilli (*P. aeruginosa, Acinetobacter baumannii*, and *Stenotrophomonas maltophilia*) (40%) followed by Enterobacteriaceae (29%) and *Staphylococcus aureus *(21%).

**Table 2:** Microorganisms responsible for the first episode of ventilator-associated pneumonia

	*n *(% of VAPs)
Gram-negative bacilli	
*Pseudomonas aeruginosa*	33 (34)
*Escherichia coli*	11 (11)
*Enterobacter *sp.	10 (10)
*Proteus mirabilis*	7 (7)
*Stenotrophomonas maltophilia*	5 (5)
*Haemophilus *sp.	3 (3)
*Klebsiella *sp.	4 (4)
*Serratia *sp.	3 (3)
Others	5 (5)
Gram-positive cocci	
Methicillin-sensitive *Staphylococcus aureus*	12 (12)
Methicillin-resistant *Staphylococcus aureus*	9 (9)
Others	10 (10)

### Clinical outcomes

#### Mortality

**Figure 3:**
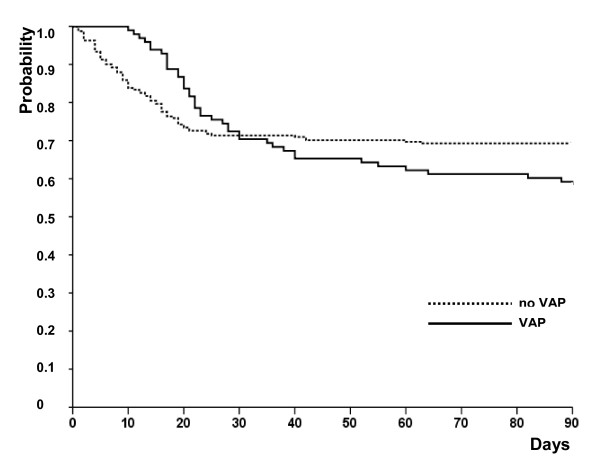
**Probability of survival through day 90 in patients with and without ventilator-associated pneumonia**.

The median time from VAP diagnosis to death was 8.5 days (IQR, 5.5 to 17 days). Of the 98 patients with VAP, 41 (41.8%; CI, 32.6% to 51.7%) died in the ICU, compared with 74 (30.7%; CI, 25.2% to 36.8%) of the 241 patients without VAP (Table 3 and Figure 3) (*P *= 0.05). In patients with early-onset VAP, mortality was 53.9% (14 of 26 patients; CI, 35.5% to 71.3%), compared with 37.5% (27 of 72 patients; CI, 27.2% to 49.1%) in patients with late-onset VAP (*P *= 0.15).

**Table 3:** Main outcome variables

	VAP % (95% CI) **no./total no**.	No VAP % (95% CI) **no./total no**.	Relative risk (95% CI)	*P *value
**Outcome**				
Primary				
ICU mortality	41.8 (32.6-51.7) 41/98	30.7 (25.2-36.8) 74/241	1.36 (1.00-1.84)	0.05
Secondary				
28-Day mortality	26.5 (18.8-36.0) 26/98	29.0 (23.7-35.1) 70/241	0.91 (0.62-1.34)	0.64
90-Day mortality	41.8 (32.6-51.7) 41/98	33.6 (27.9-39.8) 81/241	1.24 (0.93-1.67)	0.15
Ventilator-free days from day 1 to day 28	0 (0-10)	14 (0-21)		0.0001
Ventilator-free days from day 1 to day 90	50.5 (0-70)	76 (0-83)		0.0001
ICU-free days from day 1 to day 28	0 (0-0)	4 (0-16)		0.0001
ICU-free days from day 1 to day 90	32 (0-62)	66 (0-78)		0.0001
Days without failure of circulatory, coagulation, hepatic, and renal organs from day 1 to day 28	14.3 ± 10.4	14.0 ± 10.8		0.77

±, means ± SD. Differences between groups were assessed by using Student *t *test, Wilcoxon test, or χ^2 ^analysis. The number of ventilator-free days is the mean number of days from day 1 to day 28 or day 90 with spontaneous breathing for at least 48 consecutive hours. CI, 95% confidence interval; NMBA, neuromuscular blocking agent.

To determine whether VAP was an independent risk factor for dying during the ICU stay in ARDS patients, we performed a multiple logistic regression and a Cox analysis by using a multistate model. As mentioned in Table 4, age, male sex, plateau pressure on inclusion, SOFA on inclusion, McCabe score, the systematic use of NMBA, and the occurrence of VAP were entered into the logistic regression model. The occurrence of a VAP was not independently associated with the risk of ICU death. With the multistate model, and after controlling for the same risk factors, we were able to confirm that the occurrence of a bacterial VAP was not associated with the risk of ICU death (HR, 0.25; 95% CI from 0.003 to 23.5; *P *= 0.55). We conducted a *post hoc *analysis excluding the patients who were not ventilated for at least 9 days after inclusion (the first death in the VAP group occurred at day 10). Survival was higher in the group of ARDS patients not developing a VAP (*P *= 0.038 by log-rank test). However, VAP was not associated independently with death with a Cox regression analysis with the same risk factors (*P *= 0.055).

**Table 4:** Factors associated with ICU death by logistic regression

	ICU survivors *n *= 224	ICU nonsurvivors *n *= 115	Univariate unadjusted *P *value	OR (CI 95%)	Multivariate adjusted *P *value
Age (years), mean ± SD	55 ± 15	64 ± 14	0.0001	1.045 (1.026-1.063)	0.0001
Males, *n *(%)	151 (67.4)	87 (75.7)	0.12	1.469 (0.842-2.565)	0.18
Plateau pressure on inclusion (cm of water), mean ± SD	24.5 ± 4.7	25.2 ± 4.1	0.15	1.057 (1.000-1.116)	0.048
SOFA on inclusion, mean ± SD	9.6 ± 3.3	10.7 ± 3.5	0.006	1.132 (1.051-1.218)	0.001
NMBA, *n *(%)	125 (55.8)	52 (45.2)	0.065	0.568 (0.347-0.930)	0.024
VAP, *n *(%)	57 (25.4)	41 (35.7)	0.05	1.410 (0.833-2.386)	0.20
McCabe nonfatal, *n *(%)	179 (79.9)	79 (68.7)	0.022	0.592 (0.339-1.034)	0.066

CI 95%, 95% confidence interval for odds ratio; McCabe nonfatal, no death within 5 years; NMBA, neuromuscular blocking agent; OR, odds ratio; SOFA, sequential organ-failure assessment; VAP, ventilator-associated pneumonia.

#### Ventilator-free days

**Figure 4:**
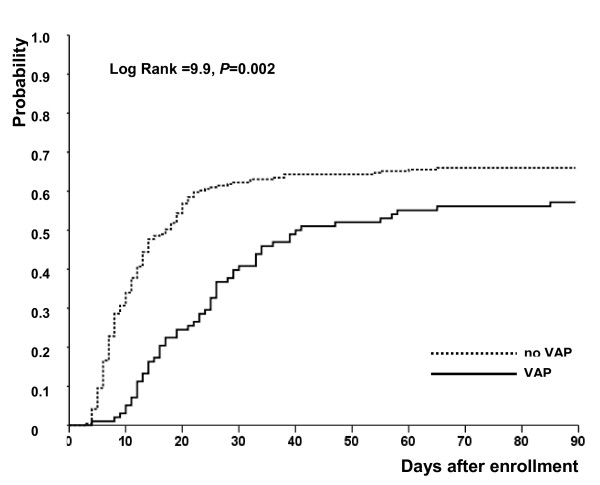
**Probability of breathing without assistance from the day of inclusion to day 90**.

As indicated in Table 3, the numbers of ventilator-free days from day 1 to day 28 and to day 90 were significantly larger in patients without VAP than in patients with VAP. The median duration of mechanical ventilation was longer in patients with VAP who were alive on day 90. As shown in Figure 4, patients without VAP were disconnected earlier from the ventilator.

#### Adequacy of empiric antibiotic treatment

Empiric treatment was adequate in 95% of patients. The mean duration of antibiotic treatment for VAP was 10.4 ± 5.2 days overall and 11.3 ± 5.2 days in patients alive at ICU discharge.

### Risk factors for ventilator-associated pneumonia

By univariate analysis (Table 5), male sex and transport out of the ICU were strongly associated with VAP. Both consciousness alterations and enteral nutrition showed trends toward associations with VAP. In contrast, VAP was not associated with NMBA use, stress-ulcer prophylaxis (proton-pump inhibitors, sucralfate, H_2_-blockers, or antacids), ARDS severity, or admission SAPS II score. All factors listed in Table 6 were entered into the multistate model. Male sex, baseline Glasgow Coma Scale score, tracheostomy (protective), enteral nutrition (protective), and the use of a subglottic secretion-drainage system (protective) were independently associated with VAP.

**Table 5:** Risk factors potentially associated with VAP

	VAP	No VAP	*P *value
	Number (%) or mean ± SD	Number (%) or mean ± SD	
Age (years)	60 ± 14	57 ± 16	0.71
Male sex	79 (81)	159 (66)	0.006
Glasgow Coma Scale score on admission	10.9 ± 4.9	11.8 ± 4.2	0.09
SAPS II on inclusion	49 ± 15	49 ± 15	0.47
PaO_2_:FIO_2 _on inclusion	106 ± 33	111 ± 40	0.48
Respiratory system compliance on inclusion (ml/cm of water)	30 ± 10	32 ± 11	0.72
Received NMBA during the first 48 hours	48 (49)	129 (54)	0.36
Stress-ulcer prophylaxis^a^	81 (87)	206 (88)	0.80
Enteral nutrition^a^	80 (86)	179 (77)	0.09
Emergency reintubation^a^	18 (19)	28 (12)	0.11
Tracheostomy^a^	30 (32)	53 (23)	0.15
Transport out of the ICU^a^	42 (45)	66 (28)	0.009
Subglottic secretion drainage^a^	11 (11)	44 (18)	0.14
Selective digestive decontamination^a^	8 (9)	18 (8)	0.82
Corticosteroids for ARDS^a^	22 (22)	43 (18)	0.33
Vasopressor^a^	91 (93)	215 (89)	0.31
Renal replacement therapy	37 (38)	81 (34)	0.47
Prone position^a^	21 (21)	64 (27)	0.46

^a^Prior VAP (VAP group) or duration of mechanical ventilation + 2 days for the no-VAP group; NMBA, neuromuscular blocking agents; SAPS II, Simplified Acute Physiology Score II; VAP, ventilator-associated pneumonia.

**Table 6:** Risk factors associated with the occurrence of bacterial VAP

	Hazard ratio	95% confidence interval for hazard ratio		*P*
		Lower	Upper	
Male sex	2.39	1.39	4.14	0.002
SAPS II on inclusion	0.99	0.97	1.00	0.14
Glasgow Coma Scale score on admission	0.93	0.88	0.98	0.01
PaO_2_:FIO_2 _on inclusion	0.99	0.99	1.00	0.54
Respiratory system compliance on inclusion	0.98	0.96	1.01	0.15
Received NMBA for 48 hours	1.03	0.69	1.54	0.88
Emergency reintubation	1.14	0.67	1.92	0.63
Tracheostomy	0.45	0.27	0.74	0.001
Transport out of the ICU	1.07	0.69	1.64	0.77
Enteral nutrition	0.56	0.33	0.97	0.04
Subglottic secretion drainage	0.52	0.27	0.99	0.05

Hazard ratios are for ventilator-associated pneumonia (VAP) versus no VAP by using the multistate model. NMBA, neuromuscular blocking agent.

## Discussion

Our study has three main findings. First, bacterial VAP was diagnosed in approximately one third of patients with severe ARDS. Second, VAP was associated with a higher crude ICU mortality rate. However, no effect of VAP on ICU mortality was found after adjustment. Third, male sex and baseline Glasgow Coma Scale score were associated with VAP in our patients with severe ARDS, whereas tracheostomy, enteral nutrition, and the use of a subglottic secretion-drainage system were protective.

### Study rationale

Although several [6-10] studies evaluated the incidence of VAP in patients with ARDS, only one [8] sought to identify specific risk factors for VAP, and all but one [8] used a single-center design. These studies included 30 to 134 ARDS patients (Table 7). Much more important, they were performed before the widespread use of lung-protective ventilation. To our knowledge, the epidemiology and outcomes of VAP have not been evaluated in ARDS patients receiving a strictly standardized protocol of lung-protective mechanical ventilation [12]. This standardization is important, as recent evidence indicates that cyclic stretching of lung cells promotes bacterial growth [11], suggesting that variations in the ventilation strategy may affect the risk of VAP.

**Table 7:** Main studies related to the epidemiology of VAP in ARDS patients

	Patients, number	Multicenter	Standardized MV	VAP incidence, %	ICU mortality, %	ICU mortality VAP (%)/no VAP, (%)
Sutherland *et al. *(8)	105	No	No	15	44	37.5/45
Delclaux *et al. *(6)	30	No	No	60	83	78/92
Chastre *et al. *(5)	56	No	No	55	61	52/72
Maduri *et al. *(9)	94	No	No	43	-	-
Markowicz *et al. *(7)	134	Yes	No	23	58	57/59
VAP-ACURASYS	339	Yes	Yes	29	34	42/31^a^

MV, mechanical ventilation; VAP, ventilator-associated pneumonia; ^a^*P *< 0.05

### Incidence of ventilator-associated pneumonia

None of the patients received new antibiotics before BAL or mini-BAL. Thus, the 28.9% incidence probably reflects the incidence of bacterial VAP in our ARDS patients, even if some suspicions were unconfirmed (maybe some false-negatives existed). The main difference between our study and earlier studies [6-10] is that the patients received lung-protective mechanical ventilation according to a strict protocol.

### Mortality and ventilator-associated pneumonia

The excess mortality potentially associated with VAP in patients with severe ARDS is difficult to assess, because many factors may contribute to death in such patients. The management of ARDS has changed over the last 15-year period. Lung-protective mechanical ventilation is now the standard of care. This change may contribute to explaining the differences in ICU mortality between our study (41.8% versus 30.7% with and without VAP, respectively) and previously published studies [6-8] (52% to 78% versus 59% to 92% with and without VAP, respectively), which occurred despite similar baseline severity scores (Table 7). However, improvements have occurred in general ICU care and mortality in many other critical illnesses during recent years.

### Risk factors for VAP

Only male sex and the admission Glasgow Coma Scale score were independently associated with an increased risk of developing a bacterial VAP in our patients with severe ARDS. In a study of 5,081 patients, Combes *et al. *[19] found that nosocomial pneumonia was more common in men than in women (51% versus 44%; *P *= 0.01). In a large US database including 9,080 patients, male gender was an independent risk factor for VAP. Differences in VAP risk between men and women may be related to differences in sex hormones [20], to sex-related polymorphisms affecting immune responses to bacterial agents [21], to differences in the distribution of pathogens responsible for infections, to differences in chronic comorbidities [22], and/or to differences in the level of care [23]. Severity of illness, and most notably neurologic failure [24-29], is associated with an increased risk of VAP. Finally, routine NMBA use during the early phase of ARDS was not associated with the risk of VAP. This is in contrast with some previous studies [25,30,31], in which NMBA use (for whatever duration) in nonselected mechanically ventilated ICU patients was associated with a higher risk of VAP.

### Study limitations

As stated in the ACURASYS study report [12], only 339 of 1,326 patients with severe ARDS assessed for eligibility were included. However, the vast majority of the remaining 987 patients had exclusion criteria. The strictly standardized ventilation protocol and strategy for VAP diagnosis are major strengths of our study. Viral pneumonia was not evaluated, as some of the participating centers did not routinely perform viral studies. A study of the impact of viral infection on outcomes of ARDS patients might be of interest.

## Conclusions

In those with severe ARDS, patients ventilated according to a standardized lung-protective strategy, the development of VAP was associated with a higher risk for dying in the ICU. However, no relation to ICU death was found after adjustment.

## Key messages

• In severe ARDS patients ventilated according to a strictly standardized lung-protection strategy, VAP increased crude ICU mortality.

• However, after adjustment, VAP did not remain associated with ICU mortality.

• Factors independently associated with an increased risk of acquiring VAP were male sex and admission Glasgow Coma Scale score.

## Abbreviations

ARDS: acute respiratory distress syndrome; VAP: ventilator-associated pneumonia; NMBA: neuromuscular blocking agent; PaO_2_:FIO_2_: ratio of partial pressure of arterial oxygen to fraction of inspired oxygen; BAL: bronchoalveolar lavage; (CFU)/ml: colony-forming units per milliliter; SAPS II: Simplified Acute Physiology Score; SOFA: sequential organ failure assessment.

## Competing interests

Glaxo-SmithKline France provided the cisatracurium and placebo for the study. Glaxo-SmithKline France gave 30 k€ for the ACURASYS study (main investigator, Laurent Papazian).

## Authors' contributions

LP, JMF, AR, DP, and FV were responsible for study concept and design; AG, GP, SJ, JMA, and EA, acquisition of the data; MF, KB, PA, JMF, and LP, analysis and interpretation of the data; JMF, LP, and AR, drafting of the manuscript; and EA, critical revision of the manuscript. All authors read and approved the final manuscript.

## Supplementary Material

Additional file 1 **Multistate analysis procedure**. Description of the multistate model and syntax used in the mstate package 0.2.6 of R development Core Team (2010). Click here for file
