# Supplementary material for: Ventilator-associated pneumonia and ICU mortality in severe ARDS patients ventilated according to a lung-protective strategy
Source: Crit Care. 2012 Apr 18;16(2):R65. doi: 10.1186/cc11312 (PMC3681394; doi:10.1186/cc11312)
Supplement: Additional file 1 — Multistate analysis procedure. Description of the multistate model and syntax used in the mstate package 0.2.6 of R development Core Team (2010). [file cc11312-S1.DOCX]

**Description of the multistate model**

State 2

Discharged alive

State 1

VAP

State 0

ARDS

State 3

ICU death

**Figure E1: Description of the multistate model. The boxes indicate all possible states and the arrows possible transitions for each ARDS patient during the ICU stay.**

**Syntax**

**> library(foreign)**

*To read a file in other format into a data frame*

**> library(mstate)**

*To load mstate package*

**> packageDescription("mstate", fields="Version")**

*To obtain the version of mstate used*

**> vignette("Tutorial")**

*Function vignette allows to get a tutorial of the mstate package, PDF version of vignette can be viewed within R.*

**> mem20z=read.spss("c:/mem/mem20z.sav")**

*To read spss binary file mem20z.sav*

**attach(mem20z)**

*The database is attached to the R search path*

*The first step in a multi-state model analysis is to set up the transition matrix :*

**tmat <- trans.illdeath(names = c("INC", "VAP", "death"))**

*To obtain more easily transition matrix (tmat), we used a built-in function called trans.illdeath, which is pre-defined for common multi-state models (competing risks model and the illness-death model). “Names” is a character vector containing the names of the states****.***

*The specified covariates that are to be retained in the dataset in long format named covs.*

**covs <- c("age", "sex", "NMBA", "SOFA", "McCabe" , "Plateau pressure")**

*mstate data preparation :*

**msbmtz <- msprep(time = c(NA, "VAPtime", "fVAPtime"), status = c(NA,"VAPstatut", "fVAPstatut"), data=mem20z, trans=tmat, keep=covs)**

*In the msprep function, the time and status arguments specify the column names in the dataset mem20z corresponding to the number of states in the multi-state model. Since all the patients start in state 1 at time 0, the time and status arguments corresponding to the first state do not really have a value. In such cases, the corresponding time and status value is NA.*

**events(msbmtz)**

*Gives the number of observed transitions*

*For regression purposes, we now add transition-specific covariates to the dataset. We will append the expand covariates to the dataset*

**msbmtz <- expand.covs(msbmtz, covs, append = TRUE, longnames = FALSE)**

*After having prepared the data in long format, estimation of covariate effects using Cox regression is straight forward using the coxph function of the survival package. The names of expanded covariates are cov.1, cov.2,… The extension .i refers to transition number i. In order to distinguish transition 2 from transition 3, we introduced a time-dependent covariate named VAP that indicated whether or not VAP has already occurred. For transition 2 (INC🡪death) the value of VAP equals 0, while for transition 3 (VAP🡪death) the value of VAP equals 1.*

**msbmtz$pn <- 0**

**msbmtz$pn[msbmtz$trans == 3] <- 1**

**c2z <- coxph(Surv(Tstart, Tstop, status) ~ age.1 + age.2 + age.3 + NMBA.1 + NMBA.2 + NMBA.3 + McCabe.1 + McCabe.2 + McCabe.3 + SOFA.1 + SOFA.2 + SOFA.3 + Plateaupressure.1 + Plateaupressure.2 + Plateaupressure.3 + sex.1 + sex.2 + sex.3 + VAP + strata(to), data = msbmtz, method = "breslow")**

**summary(c2z)**

*To get the final results of the model fitting.*

***For the present study, the hazard ratio of VAP (0.245647) and its p-value (0.5461) indicated that there was no effect on VAP on ICU death.***
